# Simulated and Experimental Investigation of the Mechanical Properties and Solubility of 3D-Printed Capsules for Self-Healing Cement Composites

**DOI:** 10.3390/ma14164578

**Published:** 2021-08-15

**Authors:** Se-Jin Choi, Ji-Hwan Kim, Hyojin Jeong, Ja-Sung Lee, Tae-Uk Lim, Haye Min Ko, Sung Hoon Kim, Wonsuk Jung

**Affiliations:** 1 Department of Architectural Engineering, Wonkwang University, 460 Iksan-daero, Iksan 54538, Korea; csj2378@wku.ac.kr (S.-J.C.); 3869kjh@naver.com (J.-H.K.); 2 Department of Chemistry, Wonkwang University, 460 Iksan-daero, Iksan 54538, Korea; vldkshrhnt@naver.com; 3 Department of Electronics Convergence Engineering, Wonkwang University, 460 Iksan-daero, Iksan 54538, Korea; grdltkd4@wku.ac.kr; 4 School of Mechanical Engineering, Chungnam National University, 99 Daehak-ro, Yuseong-gu, Daejeon 34134, Korea; taewook9409@naver.com; 5 Department of Chemistry & Wonkwang, Institute of Material Science and Technology, Wonkwang University, 460 Iksan-daero, Iksan 54538, Korea; 6 Department of Electronics Convergence Engineering & Wonkang, Institute of Material Science and Technology, Wonkwang University, 460 Iksan-daero, Iksan 54538, Korea

**Keywords:** 3D-printed capsule, mechanical property, solubility, cement composite, compressive strength

## Abstract

In the concrete industry, various R&D efforts have been devoted to self-healing technology, which can maintain the long-term performance of concrete structures, which is important in terms of sustainable development. Cracks in cement composites occur and propagate because of various internal and external factors, reducing the composite’s stability. Interest in “self-healing” materials that can repair cracks has led researchers to embed self-healing capsules in cement composites. Overcoming the limitations of polymer capsules produced by chemical manufacturing methods, three-dimensional (3D) printing can produce capsules quickly and accurately and offers advantages such as high material strength, low cost, and the ability to fabricate capsules with complex geometries. We performed structural analysis simulations, experimentally evaluated the mechanical properties and solubility of poly(lactic acid) (PLA) capsules, and examined the effect of the capsule wall thickness and printing direction on cement composites embedded with these capsules. Thicker capsules withstood larger bursting loads, and the capsule rupture characteristics varied with the printing angle. Thus, the capsule design parameters must be optimized for different environments. Although the embedded capsules slightly reduced the compressive strength of the cement composites, the benefit of the encapsulated self-healing agent is expected to overcome this disadvantage.

## 1. Introduction

Recently, interest in sustainable development has greatly increased, and various R&D activities focused on sustainable development are being conducted in the concrete industry, which consumes a great deal of cement and aggregate. In particular, extending the service life of concrete avoids waste and conserves materials. As a cement composite, concrete is a low cost, easily producible material but has disadvantages such as low tensile strength and brittleness [1,2,3,4]. Various factors can cause cracks to form in cement composites, which then propagate because of both internal and external deterioration factors, thereby reducing the stability of the cement composites. Research on cement composites that can “self-heal” cracks has increased in recent years [5,6,7], and many studies have proposed the use of self-healing capsules embedded in cement composites to heal such cracks. Wiktor et al. [5] investigated the crack-healing potential of a specific self-healing agent embedded in porous expanded clay particles and found that cracks of up to 0.46 mm in width could heal in bacterial concrete; however, after immersion in water for 100 days, only cracks of up to 0.18 mm in width healed in the control specimens. Meanwhile, Wang et al. [6] investigated the viability of microencapsulated spores on mortar samples. The healing ratio of these bio-microcapsule specimens was higher than that of the specimens without bacteria. The maximum width of the cracks healed in the bacterial concrete specimens was approximately four times that of the non-bacterial series. Furthermore, Hu et al. [7] evaluated the self-healing properties of glass capsules containing a one-component polyurethane healing agent, which they embedded in concrete. They found that a mixture of acetone and polyurethane in a mass ratio of 1:5 exhibited low viscosity and surface tension and produced the strongest healing effect.

Many studies have used glass to encapsulate healing agents [8,9,10,11,12,13,14,15]; however, these glass capsules cannot withstand the concrete mixing process without additional protection in the form of a cement paste or a metallic wire [8,16,17]. Therefore, as an alternative to glass, polymeric materials such as gelatin and polyurethane have been used to produce capsules [8,18,19,20,21,22,23,24,25,26] because these materials can withstand the mixing process and are more suited to satisfying capsule property requirements [8,27]. Most capsules are fabricated via chemical manufacturing methods and are encapsulated in polymer form; however, such methods exhibit remarkably low capsule reproducibility, and the resultant capsules suffer from poor shape uniformity and low thicknesses, making them structurally weak. 

In addition, an extruded cementitious capsule has been proposed to solve this problem [28,29,30]; however, it has a limited shape and it is difficult to homogeneously mix in the cement matrix.

To overcome these problems, we propose a three-dimensional (3D)-printed capsule. The 3D printing of polymer-based materials can quickly manufacture relatively accurate models, and this technology offers advantages such as high material strength, low printing costs, and the realization of complex geometries [31,32,33,34]. In addition, 3D printing offers very high reproducibility and homogeneity, along with many degrees of freedom in terms of the shape. A study case for applying a vascular type structure manufactured using 3D-printing technology in self-healing concrete has also been reported [35,36,37,38]. Several 3D-printing methods have been developed, including stereolithography, powder bed fusion, fused deposition modeling (FDM), inkjet printing, and contour crafting [32]. The FDM method is widely used for its high speed, low cost, and precision [32]. 

In addition, polymeric materials such as acrylonitrile butadiene styrene (ABS) and poly(lactic acid) (PLA) are widely used as filaments in 3D printers. ABS is a representative thermoplastic resin product, whereas PLA, which exhibits a low melting point and good moldability, is an environmentally friendly and biodegradable polymer. PLA degrades in nature via ester hydrolysis to produce lactic acid and oligomers. These biocompatible degradation products are not only metabolized by the human body but also undergo microbial conversion. Owing to this usefulness of PLA, some research on the interaction of bacteria-based PLA and lactate-derived capsules with cement matrix was disclosed [39,40]. Additionally, in the context of 3D printing, some research groups have investigated the stability of the synthesized polymers by examining the effects of solubility, temperature, and pH on their degradation. However, very few studies have examined the 3D-printing applications of such polymers; thus, the properties of PLA-based 3D-printed self-healing capsules must be investigated [41,42,43]. 

In this study, we manufactured 3D-printed capsules using the FDM method to overcome the drawbacks of capsules manufactured by chemical techniques. We performed structural analysis simulations and evaluated the mechanical properties and solubility of various 3D-printed capsules before embedding them in cement composites to investigate the effect of their wall thickness and printing direction on their applicability in self-healing cement composites

## 2. Materials and Methods

### 2.1. 3D Printing the Capsules 

PLA was selected as the material for fabricating the capsules. Using a 3D printer (Brule Ultimaker S3, BRULE, Seoul, Korea) with a 0.25 mm nozzle, we fabricated three types of capsules from 2.85-mm-diameter PLA filaments, which were constructed into layers. In all three cases, the capsule diameter was fixed at 15 mm, the layer height was 0.07 mm, infills were 100%, and the nozzle temperature was 225 °C. The wall thickness was different in each case, being set to 0.25, 0.4, or 0.8 mm. 

### 2.2. Mechanical Simulations of the Capsules

Structural analysis of the capsules was performed using Ansys software (2018 R2, ANSYS Inc. Daejeon, Korea) to obtain the design parameters for capsule fabrication. External forces up to 700 N were applied to the three types of fabricated capsules for 10 s in the vertical and horizontal directions of the stacking layer, and we analyzed the resulting stress distribution and deformation. When the angle was θ = 0°, the applied force was vertical, whereas θ = 90° indicated a horizontal force.

### 2.3. Measuring the Mechanical Properties of the Capsules

**Figure 1:**
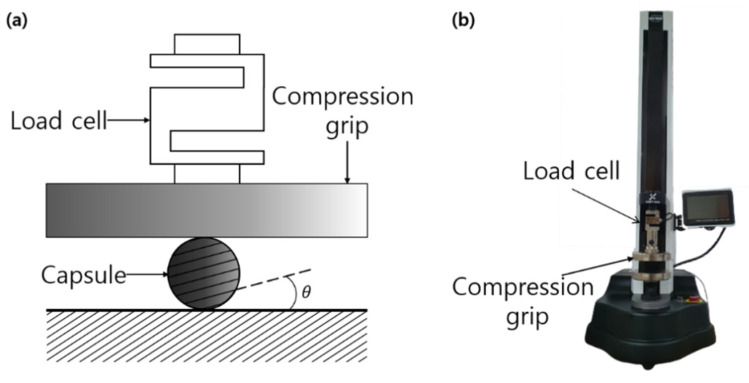
(**a**) Schematic illustration of the compression testing apparatus, (**b**) photography of the equipment and experimental setup (Test1 by Unitest-M1).

Each type of capsule was squeezed at room temperature between two parallel rigid plates moving toward each other at a constant velocity of 10 mm/min (Figure 1) using a universal testing machine (Unitest M1, TEST ONE, Busan, Korea). At the start of the experiment, the initial distance between plates was set to the initial capsule size of 2r, measured with a charge-coupled device (CCD) camera (TEST ONE, Busan, Korea) to confirm the 3D-printer-specified size.

During the test, the compressive force and capsule deformation were measured while the gap between the two plates was reduced by ∆d for each type of capsule (Figure 1). The angle between the compressive force and the additive-layer orientation of the 3D printer is an important parameter in determining the capsule properties. Therefore, we conducted these experiments with the printing angles set to either 0° or 90°, as shown in Figure 1.

### 2.4. Chemical Stability Measurements

To confirm the capsule’s sustenance and resistance against chemicals such as organic solvents, the solubility of the uncoated and lacquer- and resin-coated PLA capsules in different liquids was tested for two weeks at room temperature. Hexane, ether, ethyl acetate, methanol, an aqueous Ca(OH)_2_ solution, and a phosphate-buffered solution with pH 13 were used in this study. Various capsules were soaked in representative organic solvents and basic solution for two weeks, and then, the capsules were taken out and dried completely. The capsule weights before and after the experiment were measured, and the values were calculated by the equation for capsule weight (g) before the experiment and for dried capsule weight (g) after the experiment. Unless otherwise stated, the organic solvents were purchased from Samchun Chemical (Seoul, Korea), Aldrich (St.Louis, MO, USA), and TCI Chemicals (Tokyo, Japan).

### 2.5. Evaluating the Cement Composite Characteristics

#### 2.5.1. Materials 

ASTM type I ordinary Portland cement (Asia Cement Co., Seoul, Korea) with a fine-aggregate density of 2.6 and a fineness modulus of 2.89 was used throughout this study. Table 1 lists the chemical composition of the cement. In addition, to improve the flowability of the cement mortar, we used a water reducing agent (Seongbo Co., Jeonbuk, Korea). We embedded resin-coated 3D-printed PLA capsules with all three wall thicknesses (0.25, 0.4, and 0.8 mm) into the cement mortar; these capsules were filled with silicone oil in lieu of a healing agent to evaluate the oil outflow upon capsule rupture. 

#### 2.5.2. Mix Proportions and Specimen Preparation

**Figure 2:**
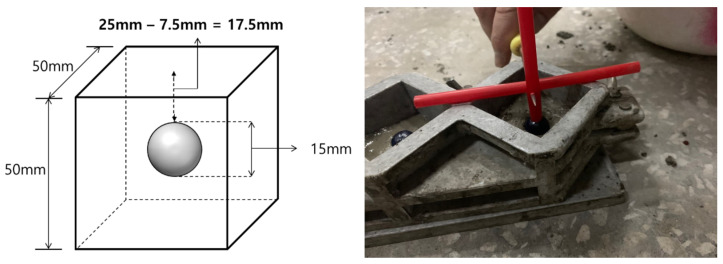
Cement mortar manufacture incorporating a 3D-printed capsule.

To examine the effect of the capsule orientation on the compressive strength of the cement composite, we inserted the capsules into the cement mortar in vertical (i.e., vertical mortar specimen, denoted by VMS; printing angle 0°) and horizontal (horizontal mortar specimen, HMS; printing angle 90°) orientations. It is expected that the capsule orientation may affect the compressive strength of the mortar owing to the printing angle. In addition, for comparison, we also prepared a plain specimen without the capsule. The cement composite specimens were mixed with water using a mechanical mixer with a water-to-cement ratio of 0.4 and filled into and 50 mm^3^ molds. Each capsule was placed at the center of the cement composite specimen (Figure 2). After 24 h, the test specimens were demolded and cured for 7 days in a constant-temperature-and-humidity curing chamber at 40 °C and a humidity of 100%. The compressive strength and unit weight of each specimen were determined according to the KS L 5105 and KS F 2462 testing standards, respectively. The strength was taken as the average value of three identical test specimens.

## 3. Results and Discussion

### 3.1. Capsule Simulation Results 

To first test the feasibility of our 3D-printed capsules, we performed a structural analysis using Ansys software to observe the stress distributions as a function of an externally applied force. The simulation parameters are summarized in Table 2. The capsules were spherical and hollow, with an outer diameter of 15 mm and a 1-mm-diameter hole at the top, and the wall thickness was set to 0.25, 0.4, or 0.8 mm. The capsule was constructed such that the PLA filament was stacked one layer at a time. In the simulation, the capsule was positioned between fixed and moveable flat plates, with a force applied to the moveable plate in the vertical direction. Simulations were conducted to compare the normal- and shear-stress cases. Upon the application of force, the normal stress acts in the vertical direction, whereas the shear stress (tangential stress) acts in the horizontal direction.

**Figure 3:**
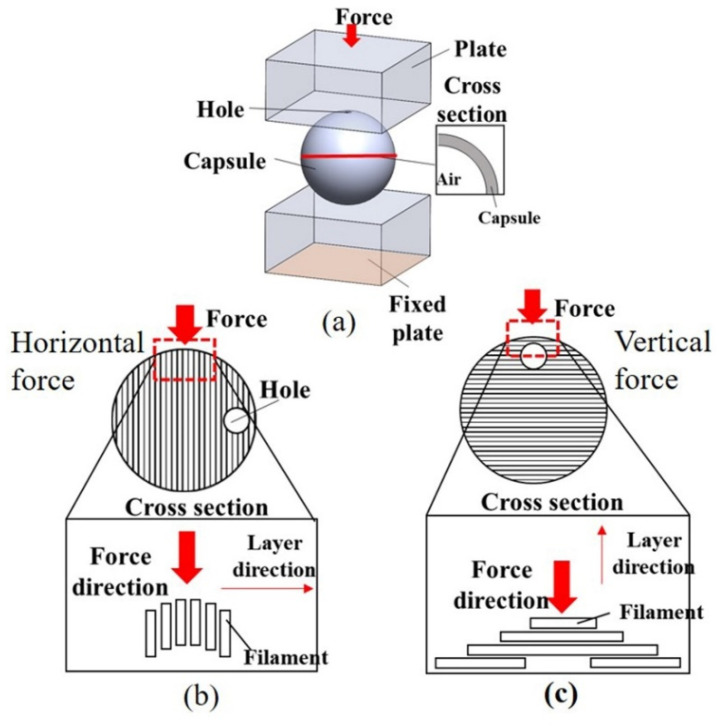
(**a**) Simulation model, (**b**) description of the horizontal force at the capsule, and (**c**) description of the vertical force at the capsule.

In the FDM 3D-printing method, filaments are stacked to produce the structure of interest, in which the deformation and destruction depend on the relationship between the direction of the applied force and the layer stacking direction; thus, both shear and normal stresses on the capsule must be analyzed. Figure 3a shows the modeling of the simulation environment, whereas Figure 3b,c depict the methods applying the horizontal and vertical forces, respectively, to the capsule. The simulations were performed with forces of 100, 400, and 700 N applied to the capsule along the horizontal and vertical directions (relative to the layer direction) to compare the stress in capsules with wall thicknesses of 0.25, 0.4, and 0.8 mm. 

**Figure 4:**
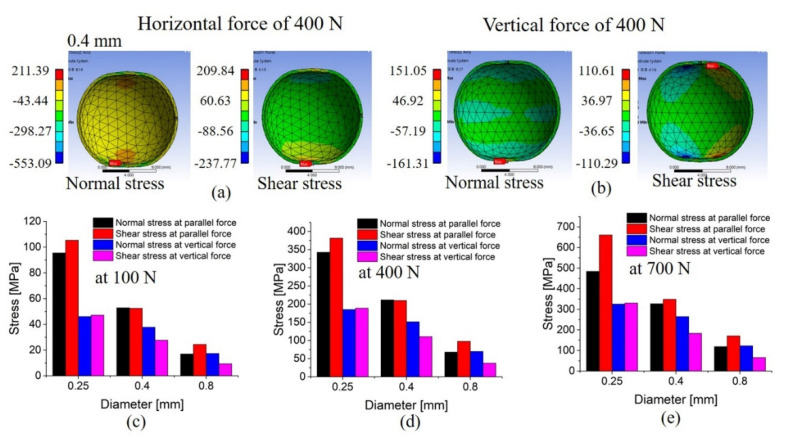
(**a**) Deformation and stress simulation of the capsule when a force of 400N is applied to the 0.4 mm capsule in the horizontal direction of the layer and (**b**) deformation and stress of the capsule when a force of 400N is applied to the 0.4 mm capsule in the vertical direction of the layer. Normal and shear stress at the thickness of 0.25, 0.4, and 0.8 mm by the applied force: (**c**) 100 N, (**d**), 400 N, and (**e**) 700 N.

Figure 4a,b show the normal- and shear-stress distributions resulting from a force of 400 N applied along the horizontal and vertical directions, respectively, to a capsule with a wall thickness of 0.4 mm. These distributions were also analyzed with applied forces of 100 and 700 N (Figure 4c,d, respectively). Although the absolute stress values vary with the external force, the stress-distribution tendencies are similar for each wall thickness. However, different wall thicknesses afford different stress-distribution patterns for a fixed external force. These results confirm that thinner capsule walls correspond to a greater stress distribution caused by the horizontal and vertical forces along the layer direction. With a wall thickness of 0.25 mm, the stress distribution due to the horizontal force was nearly twice that due to the vertical force. In other words, a thinner capsule wall ruptures more easily when a force is applied horizontally with respect to the layer. On the other hand, for thicker walls, the difference between the horizontal and vertical stress distribution decreases; thus, directionality has less influence on the rupture in such cases. 

The shear stress caused by the horizontal force on the layered capsule is large under all conditions (Figure 4c–e). Thus, our simulation analysis confirms that the stress distribution of the 3D-printed capsule is a function of the direction and magnitude of the external force as well as the capsule structural characteristics. In turn, this stress distribution governs capsule deformation and rupture. Consequently, we conducted the rupture tests of the fabricated capsules to compare the experimental results with the simulations. 

### 3.2. Mechanical Properties 

The capsule’s main functions are to protect the healing fluid contained in the capsule body and to control fluid exchange with the external medium [44]. Additionally, the capsules may be designed to release the healing fluid only under certain conditions. Therefore, the capsule’s mechanical properties (such as its rupture strength) are important design parameters critical to maintaining the capsule durability. Various methods have been proposed to measure the mechanical properties of capsules, mostly based on measuring capsule deformation under a well-defined stress [44,45,46,47,48]. A widely used technique to measure capsule deformation up to the rupture point involves squeezing the capsule between two rigid parallel plates while recording the distance between the plates as a function of the compressive force [44,45,46,47,48]. First, we prepared ten 3D-printed capsules and verified the reproducibility by statistically analyzing the fabricated capsules. When the target diameter, thickness, and layer height of the capsule design were 15, 0.4, and 0.1 mm, respectively, the observed average values of the diameter, thickness, and layer height of these fabricated capsules were 15, 0.44, and 0.99 mm, respectively. The standard deviations of these dimensions are 0, 0.02, and 0.004 mm, respectively, indicating very high reproducibility.

To implement conditions identical to those of the simulation, we 3D-printed PLA-based capsules using FDM and applied the same design parameters used in the simulations, including wall thicknesses of 0.25, 0.4, and 0.8 mm. In each case, the capsule was fixed between two plates, with the moving plate applying a compressive force to deform and rupture the capsule. 

**Figure 5:**
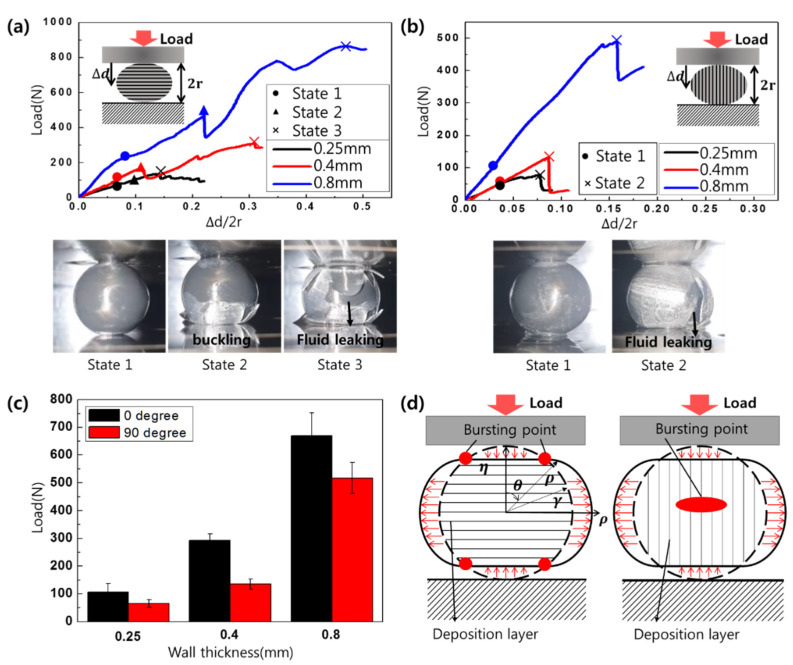
Compression test with variable capsule thicknesses for printing angles of (**a**) 0° and (**b**) 90°, respectively. (**c**) Bursting load and (**d**) schematic figures of bursting points with variable capsules having different printing angles.

As layers of PLA filaments are stacked to produce a 3D shape, the structure’s tensile strength and Young’s modulus vary with the interlayer stacking direction [46,47]. In particular, the tensile strength of the structure made with a printing angle (defined in Figure 1) of 0° is twice that of the structure made with a printing angle of 90° [46,47]. Therefore, we applied compression to capsules with various wall thicknesses at different printing angles between the applied compressive force and the layer-deposition direction to optimize the capsule design factors for each environment (Figure 5).

Figure 5a shows the deformation of capsules printed with a printing angle of 0° as a function of the compressive force. The capsules deform and rupture in three phases, regardless of their thickness. In phase 1, the capsule is compressed into an oval shape with increasing load, and in phase 2, we observe buckling in the upper section of the capsule, which is in contact with the moving plate [45]. In phase 3, the capsule eventually ruptures (“bursts”) and the healing fluid flows out of the capsule. With a printing angle of 90° (Figure 5b), the capsule deformation and rupture occurs in only two phases because bursting occurs at the interface between the deposited layer and the layer in the central area of the capsule before buckling occurs at the top. Figure 5c shows the bursting-load values for various capsule wall thicknesses when the printing angle is set to 0°: the bursting-load values are 106.5 ± 30.3 N, 293.0 ± 22.6 N, and 670.0 ± 82.0 N for capsule wall thicknesses of 0.25, 0.4, and 0.8 mm, respectively. On the other hand, for the printing angle of 90°, the bursting-load values are 66.0 ± 13.4 N, 135.7 ± 18.8 N, and 517.1 ± 55.5 N for capsule wall thicknesses of 0.25, 0.4, and 0.8 mm, respectively. Thus, the bursting loads for the printing angle of 0° are approximately 48%, 54%, and 23% lower than those compressed at 90° for capsule wall thicknesses of 0.25, 0.4, and 0.8 mm, respectively, confirming that the influence of the layer orientation is lowest when the capsule walls are thickest.

The observed difference between the two cases is due to the significantly lower vertical stress acting between the deposition layers with a printing angle of 0° relative to the corresponding values for other printing angles [46,47,48]. That is, when the capsule is compressed, the capsule section in contact with the grip deforms along the vertical direction, and the other sections expand in a direction parallel to the plate (Figure 5d) [45]. On the other hand, with a printing angle of 90°, the side portion of the capsule is subjected to stress, and thus, the fracture occurs between deposition layers under low compressive forces, causing the capsule to split in half and the stress to decrease sharply. Moreover, with a printing angle of 0°, buckling occurs at the top of the capsule, leading to bursting between the downward distortion and the deposition layer of the side-spending region boundary [49,50]. Furthermore, the crack after bursting extends along the boundaries of the laminated plane as the load gradually increases. Our analysis of the two cases indicates that both types of capsules can release the healing agent below a specific load, and the capsule wall thickness and printing angle can be optimized for different requirements. 

### 3.3. Solubility Tests 

We coated some of the 3D-printed PLA capsules with lacquer or resin to increase their stability in a variety of solvents. The solubility of the three types of the uncoated, resin-coated, and lacquer-coated capsules was tested in various solvents over two weeks at room temperature. The representative solvents were selected according to the solvent type: hexane and ether were representative non-polar solvents, and ethyl acetate and methanol were chosen as the polar aprotic and polar protic solvents, respectively. In addition, an aqueous Ca(OH)_2_ solution and a pH-13 buffer solution were selected as basic aqueous solutions owing to the strong alkalinity of the cement composite specimen. 

The solubility test results are summarized in Table 3. The uncoated PLA capsules did not dissolve in hexane, ether, methanol, or basic aqueous solutions; however, they exhibit cracking in ethyl acetate. Similar results are observed with the lacquer- and resin-coated PLA capsules. 

The capsule weight was measured before and after the solubility experiments to compare and analyze the weight change in the capsules according to the solvent used (Table 4). In ethyl acetate, significant cracking was observed for all three types of PLA capsules, and thus, the weight-change measurements were excluded. In hexane and ether solvents, the weight change lies within the error range for the coated and uncoated PLA capsules. However, in methanol, the weight of the resin-coated PLA capsules decreased slightly. Although the weight of PLA capsules in the Ca(OH)_2_ aqueous solution changed very little, interestingly, a large weight reduction was observed in the pH-13 aqueous buffer solution. Uncoated PLA capsules exhibited the greatest weight reduction, whereas the lacquer-coated PLA capsules showed the least. Therefore, we can conclude the lacquer coating protects the PLA material from dissolving in aqueous solution. We also observed that the resin coating offered PLA capsules the best protection in aqueous solutions, as the weight changes of these capsules lie well within the error range. 

### 3.4. Properties of the Cement Composites Containing Capsules

**Figure 6:**
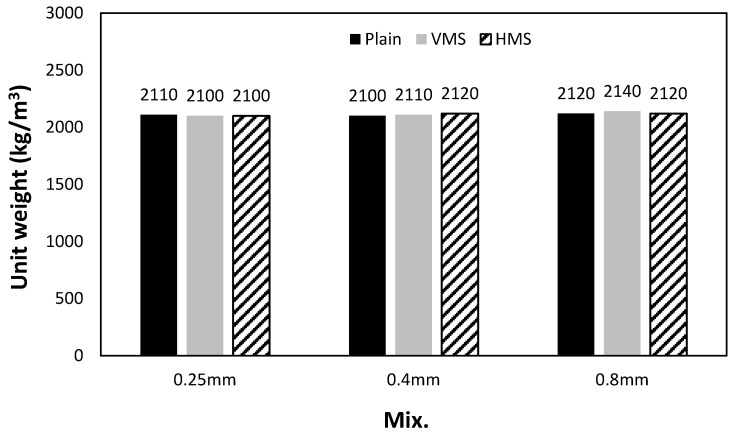
Unit weight.

**Figure 7:**
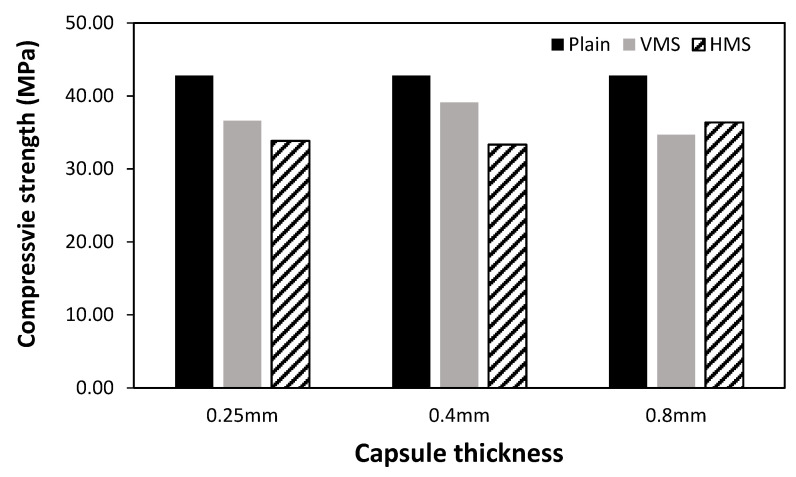
Compressive strength.

Capsules were inserted into cubic cement specimens, and the compressive strength and unit weight of the specimens were measured. In addition, each capsule was coated with resin to prevent oil leakage. Figure 6 shows the unit weights of the specimens, which range from 2100 kg/m^3^ to 2140 kg/m^3^, regardless of the capsule wall thickness. Figure 7 shows the changes in the compressive strength according to the capsule wall thickness. The specimens without capsules exhibited the highest compressive strength at ~42.8 MPa, whereas those with capsules showed slightly lower strengths. This reduction can be attributed to the air bubbles trapped under the capsule during capsule insertion and the adhesive strength between the capsule and the cement matrix.

**Figure 8:**
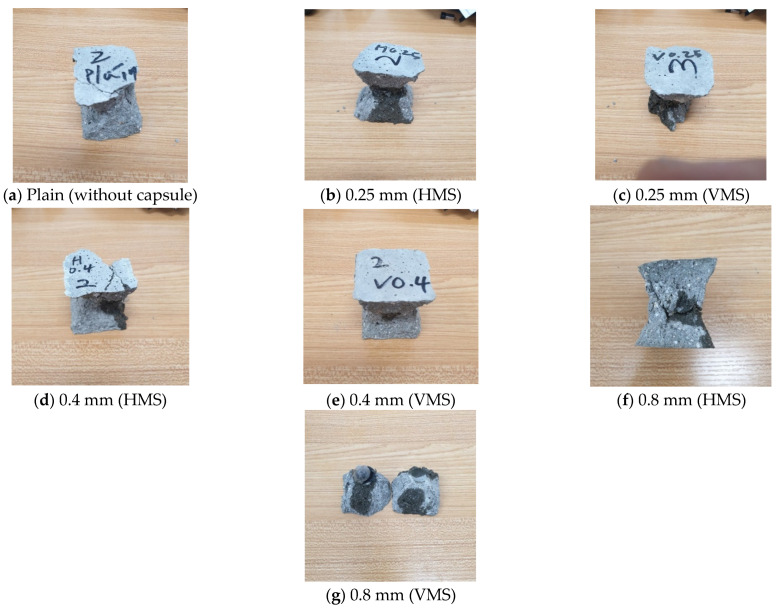
Samples after the compressive strength test.

The effect of the orientation of the capsule was also examined. For the capsule wall thicknesses of 0.25 and 0.4 mm, the VMS specimen, in which the capsule was inserted with the stacked layers in the vertical direction, was slightly stronger than the HMS specimen, with the capsule inserted in the horizontal direction. These results are similar to the trend in Figure 5c, which shows the bursting-load value of the capsule according to the printing angle. With a capsule wall thickness of 0.8 mm, the capsule orientation appeared to have to a smaller influence on the strength than that of the other thicknesses; this result can be attributed to the reduced influence of the 3D-printing stacking direction on the strength of the thickest capsule wall. Relative to that of the specimen without the capsule, the compressive strengths of the specimens embedded with a capsule ranged from ~81.0% to 91.4% for the VMS specimens and 77.8% to 84.9% for the HMS specimens. Figure 8 shows the fracture characteristics of the cement composite specimens according to the capsule wall thickness and direction. The solution within the capsule was released when the capsule ruptured. However, the 0.8-mm-thick capsule in the cement composite did not burst immediately as the maximum load was applied; continuous application was required to rupture the capsule.

## 4. Conclusions

Based on the findings of our study, we drew the following conclusions:

1. The 3D-printing method can be used to manufacture capsules with uniform shapes with a high degree of mechanical/physical reproducibility.
2. Stress simulations enabled analysis of the stress characteristics according to the stacking direction and the external force applied to the 3D-printed capsule.
3. Compressive experiments were conducted with two different printing angles and three different capsule wall thicknesses. A thicker capsule wall can withstand a larger bursting load, and the rupture characteristics vary depending on the printing angle. These results can be used to optimize the capsule design parameters for different cement environments.
4. The solubility tests on PLA capsules indicate that the capsule stability varies based on whether the solvent is non-polar, polar aprotic, polar protic, or basic. Remarkably, we observed that none of the PLA capsules (uncoated, resin-coated, and lacquer-coated) dissolve in hexane, which is a non-polar solvent. Nonetheless, resin-coated PLA capsules exhibit different properties than both uncoated and lacquer-coated capsules because of their ability to resist dissolution in all solvents.
5. Plain cement composite specimens without embedded capsules demonstrated the highest compressive strength of ~42.8 MPa, whereas the capsule-embedded specimens were somewhat weaker. In addition, with capsule wall thicknesses of 0.25 mm and 0.4 mm, the VMS specimen embedded with a vertically oriented capsule was slightly stronger than the HMS specimen with a capsule inserted horizontally.
6. Embedding 3D-printed capsules containing self-healing materials in cement composites may slightly reduce their compressive strength; however, this reduction may be outweighed by the benefit of their self-healing effects.

However, further studies are needed to establish the healing effect of the 3D-printed capsule, the size effect of the capsule, and the self-healing effect according to the type of healing agent, etc.

## Figures and Tables

**Table 1:** Chemical composition of cement.

Components	SiO_2_	Al_2_O_3_	Fe_2_O_3_	CaO	MgO	K_2_O
Cement	17.43	6.50	3.57	64.40	2.55	1.17

**Table 2:** Simulation parameters.

Properties	Value	Unit
Density	645	Kg/m^3^
Young’s modulus X, Y direction	33,000	MPa
Young’s modulus Z direction	26,000	
Poisson’s ratio YZ, XZ	0.33	-
Poisson’s ratio XY	0.28	
Shear modulus XY, YZ, XZ	9600	MPa
Tensile X, Y direction	44	
Tensile Z direction	12	
Compressive X, Y direction	−47	
Compressive Z direction	−34	
Shear XZ, YZ	30	
Shear XY	12	

**Table 3:** Solubility tests in various solvents ^a^.

Solvent	Solvent Type	PLA Capsule	Lacquer-Coated PLA Capsule	Resin-Coated PLA Capsule
Hexane	Non polar	insoluble	insoluble	insoluble
Ethyl acetate	Polar aprotic	-	-	-
Ether	Non polar	insoluble	insoluble ^b^	insoluble ^c^
Methanol	Polar protic	insoluble	insoluble ^b^	insoluble ^c^
Ca(OH)_2_ solution	Basic	insoluble	insoluble	insoluble
pH 13 buffer solution	Basic	insoluble	Insoluble ^b^	insoluble

^a^ Solubility tests in various solvents were performed for 2 weeks at room temperature. ^b^ The lacquer coating is all peeled off. ^c^ The resin coating is all peeled off.

**Table 4:** Degradation of various capsule material after solubility tests ^a^.

	Solvent	PLA Capsule	Lacquer-Coated PLA Capsule	Resin-Coated PLA Capsule
**Degree of Mass Change**	Hexane	±0.005 g	±0.031 g	±0.003 g
	Ethyl acetate	-	-	-
	Ether	±0.015 g	±0.001 g ^b^	±0.009 g ^c^
	Methanol	±0.006 g	±0.008 g ^b^	−0.076 g ^c^
	Ca(OH)_2_ solution	±0.014 g	±0.001 g	±0.016 g
	pH 13 buffer solution	−0.054 g	−0.038 g ^b^	−0.018 g

^a^ The values were calculated by equation for capsule weight (g) before test and for dried capsule weight (g) after test. ^b^ The lacquer coating is all peeled off. ^c^ The resin coating is all peeled off.

## Data Availability

Not applicable.
